# Photobiomodulation reduces gliosis in the basal ganglia of aged mice

**DOI:** 10.1016/j.neurobiolaging.2018.02.019

**Published:** 2018-06

**Authors:** Nabil El Massri, Tobias W. Weinrich, Jaimie Hoh Kam, Glen Jeffery, John Mitrofanis

**Affiliations:** a Department of Anatomy F13, University of Sydney, Sydney, NSW, Australia; b Institute of Ophthalmology, University College London, London, England

**Keywords:** Caudate-putamen complex, Substantia nigra, Interneurons, Astrocytes, Microglia

## Abstract

This study explored the effects of long-term photobiomodulation (PBM) on the glial and neuronal organization in the striatum of aged mice. Mice aged 12 months were pretreated with PBM (670 nm) for 20 minutes per day, commencing at 5 months old and continued for 8 months. We had 2 control groups, young at 3 months and aged at 12 months old; these mice received no treatment. Brains were aldehyde-fixed and processed for immunohistochemistry with various glial and neuronal markers. We found a clear reduction in glial cell number, both astrocytes and microglia, in the striatum after PBM in aged mice. By contrast, the number of 2 types of striatal interneurons (parvalbumin^+^ and encephalopsin^+^), together with the density of striatal dopaminergic terminals (and their midbrain cell bodies), remained unchanged after such treatment. In summary, our results indicated that long-term PBM had beneficial effects on the aging striatum by reducing glial cell number; and furthermore, that this treatment did not have any deleterious effects on the neurons and terminations in this nucleus.

## 1 Introduction

A characteristic feature of the central nervous system in aging is an activation of glial cells (Lynch et al., 2014, Soreq et al., 2017). For example, many previous studies have reported a marked increase in the number of glial cells due to aging, both astrocytes and microglia, across the central nervous system (Beach et al., 1989, Unger, 1998). For both types of glial cells, there is also an increase in their size and immunohistochemical expression of various markers (Beach et al., 1989, Begum et al., 2013, Conde and Streit, 2006, Cotrina and Nedergaard, 2002, Unger, 1998).

A further feature of aging is that, in contrast to the increase in glial cell number, there is a progressive loss of neurons. This loss manifests after a series of intrinsic molecular changes within the neurons leading to dysfunction and ultimately death. Furthermore, such changes render aging neurons more susceptible to insult, whether by environmental toxin or genetic mutation, such as in Alzheimer's or Parkinson's disease. In fact, aging is a major risk factor for both these neurodegenerative disorders (Balaban et al., 2005, Kujoth et al., 2005, Linnane et al., 1989, Salvadores et al., 2017).

There is general agreement that a pivotal part of the intrinsic change leading to glial and neuronal aging and death is dysfunction of the mitochondria. Mitochondria drive neuronal function by producing adenosine triphosphate and with aging, this ability diminishes. This is associated with an increase in toxic reactive oxygen species, oxidative stress, and subsequent neuronal death (Balaban et al., 2005, Kujoth et al., 2005, Linnane et al., 1989, Salvadores et al., 2017).

In view of these key features of aging, the development of treatments that target a reduction of gliosis and the protection of mitochondria in neurons have generated much interest (Chaturvedi and Beal, 2008). In this context, recent studies have shown that relatively short-term exposure to red to infrared light (λ = 600–1070 nm), or photobiomodulation (PBM), not only reduces gliosis markedly (Begum et al., 2013, El Massri et al., 2016b, El Massri et al., 2016a,) but also improves mitochondrial function (Begum et al., 2013, Eells et al., 2004, Gkotsi et al., 2014, Hamblin, 2016, Karu, 2010, Khan and Arany, 2015, Rojas and Gonzalez-Lima, 2011, Sivapathasuntharam et al., 2017), in both aging and disease.

In this study, we explored whether long-term PBM had any impact, beneficial or deleterious, on gliosis and/or neuronal survival in aging. We chose the caudate-putamen complex, or striatum, of the basal ganglia for investigation mainly because of our long-standing interest in Parkinson's disease (El Massri et al., 2016a, El Massri et al., 2016b, Shaw et al., 2010), and that it represents a central “hub” of functional neurotransmission for many other neural centers, from the cerebral cortex to the thalamus and to all the other nuclei in the basal ganglia (Parent and Hazrati, 1995). Furthermore, except for features of the dopaminergic system (Darbin, 2012), few studies have explored the glial and neuronal organization of the striatum, together with the greater basal ganglia, in aging. Indeed, no previous study has explored the effects of PBM in this key brain area in aging. To this end, we examined several cellular structures of the striatum, namely, the 2 types of glial cells (glial fibrillary acidic protein [GFAP]^+^ astrocytes and ionized calcium-binding adapter molecule 1 [IBA1]^+^ microglia), 2 types of neurons (parvalbumin [Pv]^+^ and encephalopsin [Eno]^+^), and 1 type of termination (tyrosine hydroxylase [TH]^+^). In general, by using these striatal structures as examples, we aimed to gain insight into the overall age-related changes evident in the basal ganglia and the impact after PBM.

## 2 Materials and methods

### 2.1 Subjects

Male C57BL/6 mice (n = 16) were housed on a 12-hour light/dark cycle with unlimited access to food and water. Animals were aged at 3 (young) or at 12 (aged) months old (examining animals at only 1 stage for young [3 months] and using 12 months as aged is common [e.g., Vacano et al., 2018]). All experiments were approved by the Animal Ethics Committee of the University College London and Home Office–licensed procedures conforming to the UK Animal Licence Act (1986). We had 3 groups of mice; 3m (aged 3 months, n = 5; young controls, with no PBM), 12m (aged 12 months, n = 6; old controls, with no PBM), and 12m + PBM (aged 12 months, n = 5; PBM treated).

### 2.2 Photobiomodulation

Animals in the 12m + PBM group were treated with PBM (670 nm) for 20 minutes per day. This treatment occurred in the morning. Treatment commenced when the animals were 5 months old and continued for 8 months, up until the animals reached 12 months of age. Our rationale for commencing treatment at 5 months was that at this age, mice in the wild are considered “old”, but in other respects “normal”. We hence have used fully mature mice (5 months) and mapped progress through to an older age (12 months), recording any changes to this progress with or without PBM.

### 2.3 Immunohistochemistry and cell analysis

Mice had their brains aldehyde-fixed (4% buffered paraformaldehyde), cryoprotected, and sectioned coronally using a freezing microtome (El Massri et al., 2016a, El Massri et al., 2016b, Shaw et al., 2010). Sections of striatum were incubated in normal goat serum (KPL) and then in either rabbit anti-GFAP (1:500, ab7260; Abcam; to label astrocytes), rabbit anti-IBA1 (1:1000, ab178846; Abcam; to label microglia), rabbit anti-TH (1:500, T8700; Sigma; to label dopaminergic terminals), rabbit anti-Eno (1:500, ab75285; Abcam; to label striatal neurons), or mouse anti-Pv (1:3000, P3088; Sigma; to label striatal neurons) followed by biotinylated goat anti-rabbit or anti-mouse IgG and then streptavidin-peroxidase complex (71-00-19; KPL). In addition, sections of the midbrain were incubated in anti-TH to label the dopaminergic cells that project to the striatum, and these were processed further as described previously. Finally, all sections were reacted in a 3,3′- diaminobenzidine tetrahydrochloride solution (D3939; Sigma) and then coverslipped. For controls, sections were processed as described previously except that no primary antibody was used. These control sections were immunonegative. For cell analysis, the number of immunoreactive cells in the striatum (and the midbrain) was estimated using the optical fractionator method (Stereo Investigator; MBF Science), as described previously (El Massri et al., 2016a, El Massri et al., 2016b, Shaw et al., 2010). We also measured the density of TH^+^ terminals in the striatum. Bright-field images of TH^+^ terminals were captured under standard illumination conditions for each section. Each image was then processed in an identical manner using ImageJ software (NIH). For each image, color threshold was adjusted to a set level, when the TH^+^ terminals were distinguished from background. The mean gray value was then measured for each image. The resulting values in the striatum provided a reliable and replicable measure of the density of TH^+^ terminals in each image (El Massri et al., 2016a). For comparisons in the number of cells and density of terminations between groups, a 1-way analysis of variance (ANOVA) test was performed, in-conjunction with a Tukey multiple comparison test was used (GraphPad Prism).

## 3 Results

Our results will explore the age-related changes, and the effects of PBM, in the striatum. The changes in glia, neurons, and terminal patterns will be considered separately.

### 3.1 Glia

**Fig. 1:**
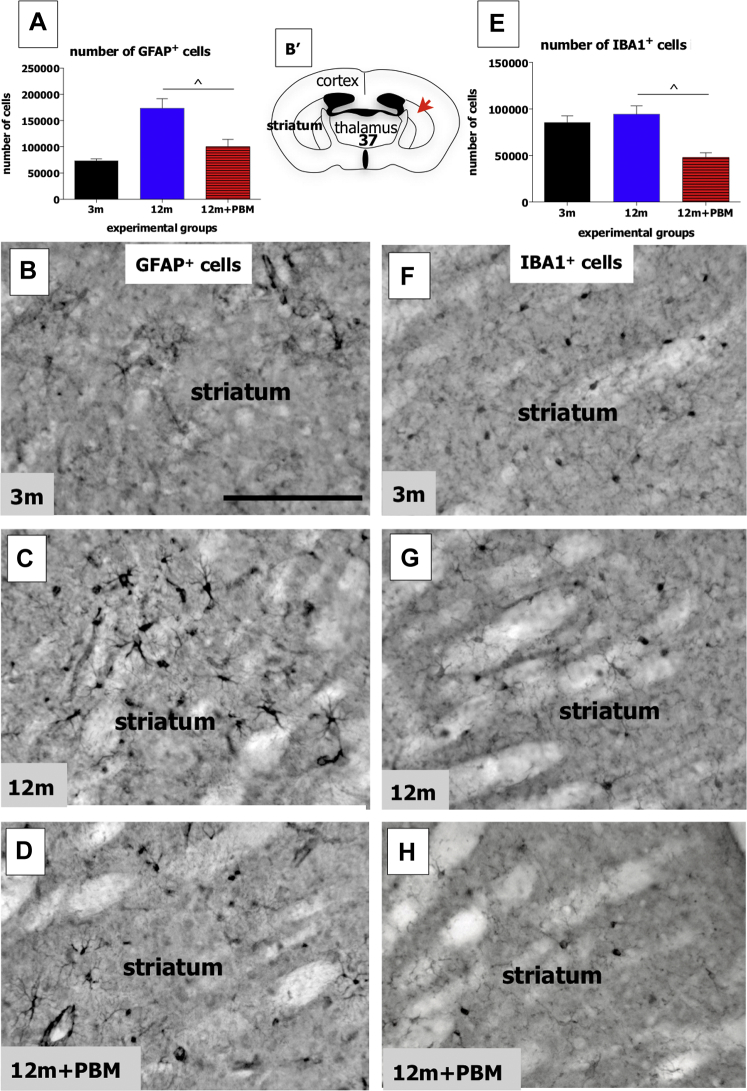
Graphs showing the total number of GFAP^+^ astrocytes (A) and of IBA1^+^ microglia (E) in the striatum of the different experimental groups. Error bars indicate SEM. The symbol (ˆ) represents significant difference (*p* < 0.01) using Tukey-Kramer multiple comparison test. Photomicrographs of GFAP^+^ astrocytes in the 3m (B), 12m (C), and 12m + PBM (D) groups and of IBA1^+^ microglia in the 3m (F), 12m (G), and 12m + PBM (H) groups. Schematic diagram of the mouse forebrain (B') adapted from a mouse atlas (Paxinos and Franklin, 2001). The red arrow indicates the approximate region where the photomicrographs of the striatum (B–D and F–H) were taken from; the bold number corresponds to the plate number in the atlas. Scale bar = 100 μm. Abbreviations: GFAP, anti-glial fibrillary acidic protein; IBA1, ionized calcium-binding adapter molecule 1; PBM, photobiomodulation; SEM, standard error mean. (For interpretation of the references to color in this figure legend, the reader is referred to the Web version of this article.)

There were marked changes evident in both types of glial cells following long-term PBM in the striatum of aged mice. Fig. 1A shows a graph of the estimated total number of GFAP^+^ astrocytes in the striatum in the different experimental groups. There were clear differences in cell number between the different groups (Fig. 1A; ANOVA: F = 14; *p* < 0.001). There was a ∼60% increase in the number of GFAP^+^ astrocytes between the 3m and 12m groups (Tukey-Kramer: *p* < 0.0001). In the 12m + PBM group, the number of astrocytes was much lower than the 12m group (Tukey-Kramer: *p* < 0.01), being similar to the 3m group (Tukey-Kramer: *p* > 0.05). In terms of morphology and overall immunoreactivity, GFAP^+^ cells of the 12m group (Fig. 1C) tended to be much larger and more strongly labeled than those of the 3m (Fig. 1B) and 12m + PBM (Fig. 1D) groups. They appeared “activated”. For the IBA1^+^ microglial cells, there were differences in cell number between the different groups also (Fig. 1E; ANOVA: F = 11; *p* < 0.001). Although there were no differences in the number of IBA1^+^ microglia between 3m and 12m groups (Tukey-Kramer: *p* > 0.05), there was a ∼50% reduction in cells between the 12m (and 3m) and 12 + PBM groups (Fig. 1E; Tukey-Kramer: *p* < 0.01). In terms of morphology, we found no differences evident among IBA1^+^ microglia of the 3m (Fig. 1F), 12m (Fig. 1G), and 12m + PBM (Fig. 1H) groups; we encountered no “activated”, amoeboid-like cells, with all cells having the classical resting-state morphology. In summary, we found that long-term PBM had a major effect on the number of glial cells in the striatum of older animals.

### 3.2 Neurons

**Fig. 2:**
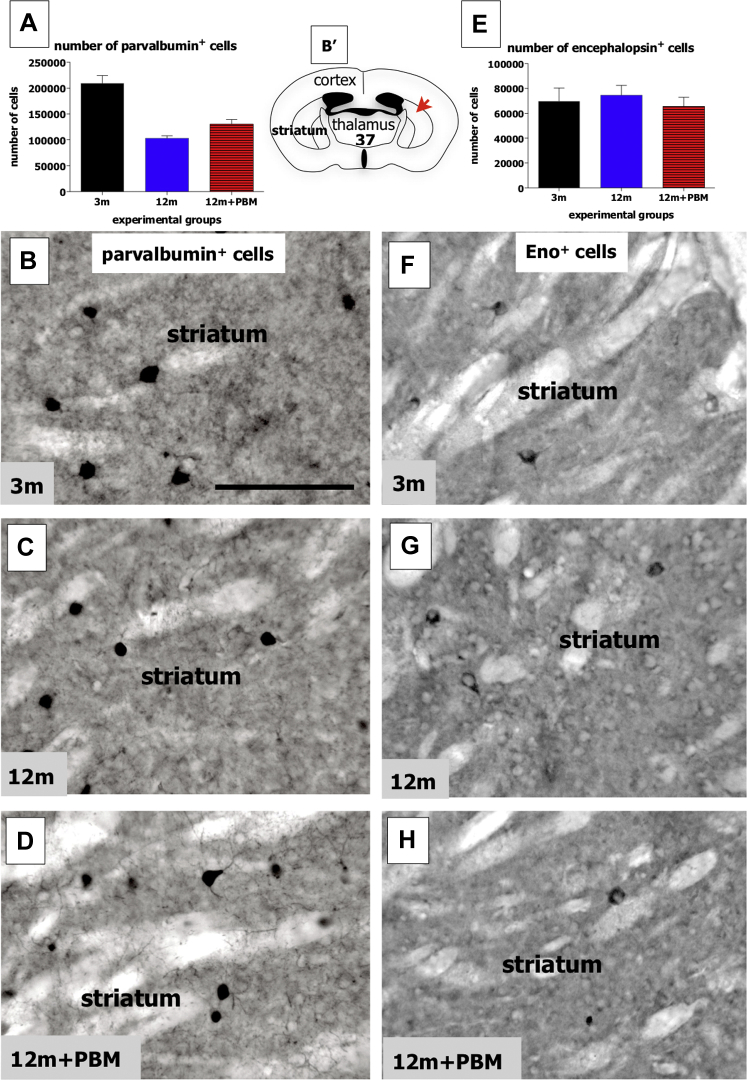
Graphs showing the total number of parvalbumin^+^ cells (A) and of encephalopsin^+^ cells (E) in the striatum of the different experimental groups. Error bars indicate SEM. Photomicrographs of parvalbumin^+^ cells in the 3m (Fig. 1B), 12m (Fig. 1C), and 12m + PBM (Fig. 1D) groups and of encephalopsin^+^ cells in the 3m (Fig. 1F), 12m (Fig. 1G), and 12m + PBM (Fig. 1H) groups. Schematic diagram of the mouse forebrain (B′) adapted from a mouse atlas (Paxinos and Franklin, 2001). The red arrow indicates the approximate region where the photomicrographs of striatum (B–D and F–H) were taken from; the bold number corresponds to the plate number in the atlas. Scale bar = 100 μm. Abbreviation: PBM, photobiomodulation; SEM, standard error mean. (For interpretation of the references to color in this figure legend, the reader is referred to the Web version of this article.)

In contrast to the findings on the glial cells, we found no major changes after long-term PBM in the 2 distinct neuronal types we examined, namely those that express Pv or Eno. For Pv^+^ cells, previous studies have reported that these form a subgroup of the GABAergic (γ-aminobutyric acid) interneurons in the rodent striatum (Kawaguchi et al., 1995). Overall, there were clear differences in the numbers of Pv^+^ cells between the experimental groups (Fig. 2A; ANOVA: F = 31; *p* < 0.0001), due mainly to the ∼50% reduction in their number between the 3m and 12m groups (Tukey-Kramer: *p* < 0.0001). There were no major differences, however, between the 12m and 12m + PBM groups (Tukey-Kramer: *p* > 0.05). In terms of morphology and overall immunoreactivity, Pv^+^ cells of the 3m (Fig. 2B), 12m (Fig. 2C), and 12m + PBM (Fig. 2D) groups were very similar. For the Eno^+^ cells, these have been localized to a group of interneurons also, but to a distinct set, namely the cholinergic interneurons (El Massri et al., 2017). Unlike the Pv^+^ cells, there were no substantial differences in the numbers of Eno^+^ cells between the experimental groups (Fig. 2E; ANOVA: F = 0.3; *p* = 0.77), in particular between the 3m and 12m groups and between the 12m and 12m + PBM groups (Tukey-Kramer: *p* > 0.05). In terms of morphology and patterns of immunoreactivity, there were no major differences evident among Eno^+^ cells of the 3m (Fig. 2F), 12m (Fig. 2G), and 12m + PBM (Fig. 2H) groups. In summary, for the 2 types of striatal interneurons we examined in this study, we found that long-term PBM had no effect on their number in older animals.

### 3.3 Terminal patterns

**Fig. 3:**
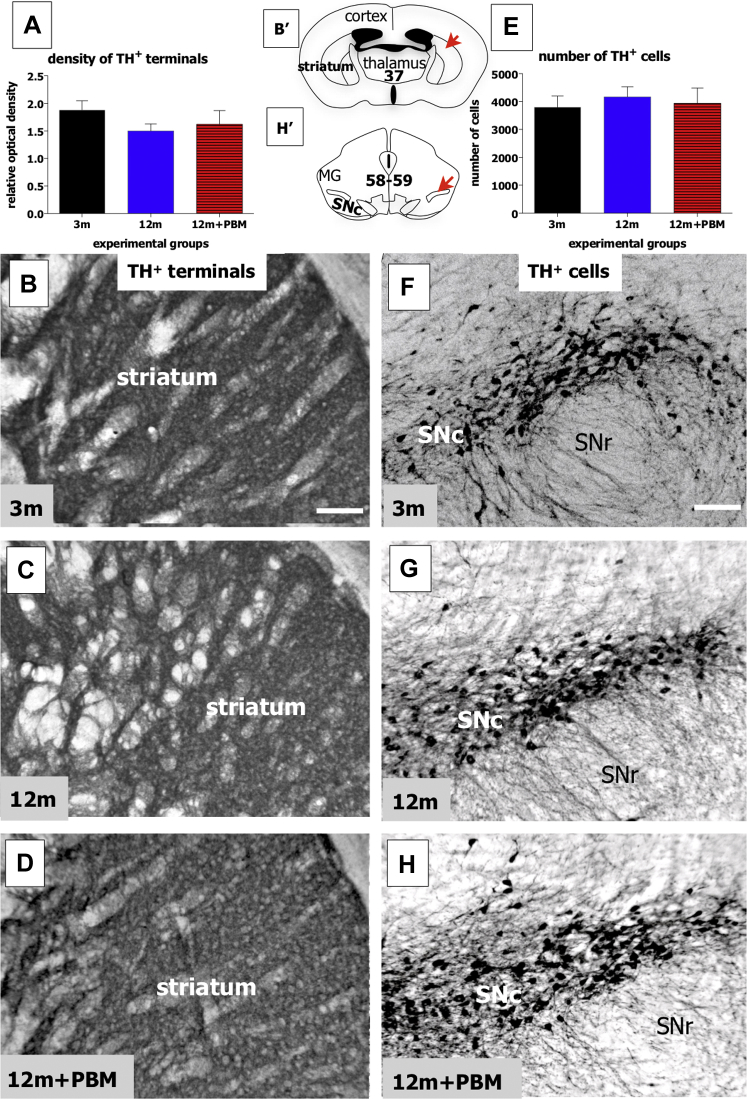
Graphs showing the mean density of TH^+^ terminals in the striatum (A) and the total number of TH^+^ cells in the SNc of the midbrain (E) of the different experimental groups. Error bars indicate SEM. Photomicrographs of striatal TH^+^ terminals in the 3m (Fig. 1B), 12m (Fig. 1C), and 12m + PBM (Fig. 1D) groups and of SNc TH^+^ cells in the 3m (Fig. 1F), 12m (Fig. 1G), and 12m + PBM (Fig. 1H) groups. Schematic diagrams of the mouse forebrain (B′) and midbrain (taken H′) adapted from a mouse atlas (Paxinos and Franklin, 2001). The red arrows in each schematic indicate the approximate regions where the corresponding photomicrographs of striatum (B–D) and SNc (F–H) were taken from; the bold number on each schematic corresponds to the plate number in the atlas. Scale bar = 100 μm. Abbreviations: TH, tyrosine hydroxylase; SNc, substantia nigra pars compacta; SNr, substantia nigra pars reticulata; PBM, photobiomodulation; SEM, standard error mean. (For interpretation of the references to color in this figure legend, the reader is referred to the Web version of this article.)

As with the 2 types of striatal interneurons, we found no substantial differences in the mean density of striatal TH^+^ terminals in the 3 experimental groups used in this study (Fig. 3A; ANOVA: F = 1.1; *p* = 0.35). In each group, namely the 3m (Fig. 3B), 12m (Fig. 3C), and 12m + PBM (Fig. 3D), strongly labeled TH^+^ terminals were seen across the nucleus, with no major zones of lighter or no labeling. We also examined the number of TH^+^ cell bodies in the substantia nigra pars compacta (SNc) of the midbrain, the main sources of the TH^+^ terminations to the striatum. Again, there were no substantial differences between the number of TH^+^ cells in the SNc in the different experimental groups (Fig. 3E; ANOVA: F = 0.2; *p* = 0.83), namely 3m (Fig. 3F), 12m (Fig. 3G), and 12m + PBM (Fig. 3H). In summary, neither aging nor long-term PBM had any effect on the dopaminergic system of mice, with no changes being recorded in the density of TH^+^ terminations in the striatum and in the number of TH^+^ cells in the midbrain SNc.

## 4 Discussion

The aim of this study was to explore the effects of long-term PBM on the glial and neuronal organizations of the striatum in aging. In general, our results indicated that in mice aged 12 months, PBM had a greater impact on reducing glial numbers than on increasing neuronal ones; and furthermore, that this treatment had no deleterious effects on either type of brain cells. The latter is a key safety issue, particularly with regard to the use of this therapy in humans. Specifically, we found a clear reduction in the number of glial cells, both astrocytes and microglia, in the striatum after long-term PBM. By contrast, the number of 2 types of interneurons, together with the density of a major terminal input to the striatum, remained unchanged after long-term PBM. These findings will be the focus of the Discussion below.

Our most striking finding was that long-term PBM generated a change in the number of glial cells in aging. These changes in number can serve as important indicators as to the overall impact of PBM in the aging and diseased brain (e.g., Beach et al., 1989, Fernagut et al., 2004, Mouton et al., 2002, Ramírez et al., 2001) and have been used by many previous authors exploring different issues and therapeutic methods in the aged and diseased neural system (e.g., Bakalian et al., 1991, Ohm et al., 1997, Pelvig et al., 2008, Fabricius et al., 2013, El Massri et al., 2016b, El Massri et al., 2016a,). For both glial cell types, astrocytes and microglia, we found fewer cells in the striatum of the 12m + PBM than in the 12m group (Fig. 1). For the astrocytes, their number in the 12m + PBM group was not only much lower than the 12m group, but it was similar to the 3m group, indicating that PBM offset almost completely the age-related astrogliosis in the striatum. The astrogliosis we found in the striatum has also been noted in other regions of the aging brain (Beach et al., 1989, Conde and Streit, 2006, Cotrina and Nedergaard, 2002, Unger, 1998) and retina (Ramìrez et al., 2001). Furthermore, PBM has been shown to reduce GFAP expression in astrocytes (and Müller cells) in the aged retina (Begum et al., 2013). It remains to be determined whether the reduction in the number of GFAP^+^ astrocytes we found in the striatum after PBM was due to a functional reduction in GFAP expression within cells or to a true reduction of the cells themselves. In line with many other studies in different systems (e.g., Brahmachari et al., 2006), we suggest that the bulk of the changes we found were due to a functional downregulation of GFAP expression. Furthermore, whether this downregulation of GFAP was in response to changes in striatal neuronal numbers or a direct impact on the astrocytes themselves is not clear (El Massri et al., 2016b). From our results, where we noted no PBM-induced changes in the number of 2 neuronal types (see below), one would assume that there was a direct impact of PBM on the astrocytes, rather than as a secondary response to a change in neuron number. However, a much more exhaustive investigation of the changes in the number of many different types of striatal neurons, well beyond the scope of this study, would establish if this is the case. For the microglia, unlike the astrocytes, we found no clear increase in their numbers between 3m and 12m groups, indicating no evidence for microgliosis in our model; furthermore, we found no activated “amoeboid-like” cells in the striatum of the 12m group. Previous studies have reported that different brain regions and the retina all show signs of microgliosis, but at different stages of aging. For example, the midbrain SNc develops microgliosis in mice aged 12 months (Schumm et al., 2012), whereas the cerebral cortex (Tremblay et al., 2012) and perhaps the retina also (Damani et al., 2011, Ma and Wong, 2016) develop microgliosis at a later stage, from 18–24 months. Following, it is likely that the striatum is one of those brain regions, like the cerebral cortex, for example, that develops microgliosis at a later stage in aging, well after 12 months of age in mice. Finally, we found that the 12 + PBM group, rather curiously, had many fewer microglia cells than the 12m, as well as the 3m, group. The significance of this finding is not clear, but we suggest that PBM may have had an inhibitory effect on microgliosis in the striatum, that this treatment served as a prophylaxis to microglial hypertrophy (Zecha et al., 2016).

In contrast to the effect on glial cells in the striatum of aged mice, our results showed that long-term PBM had no impact on the 2 types of striatal interneurons examined in this study, namely the Pv^+^ and Eno^+^ cells. For both neuronal types, there were no differences in their number between the 12m and 12m + PBM groups (Fig. 2). The result was particularly relevant for the number of Pv^+^ cells that, unlike the number of Eno^+^ cells, showed a marked decrease from the 3m to the 12m group. This reduction was not mitigated by PBM (Fig. 2).

The beneficial outcomes of PBM are thought to involve an activation of a photoacceptor, such as cytochrome c oxidase, leading to an increase in electron transfer in the respiratory chain within the mitochondria and increased adenosine triphosphate production (Begum et al., 2013, Eells et al., 2004, Gkotsi et al., 2014, Hamblin, 2016, Karu, 2010, Khan and Arany, 2015, Rojas and Gonzalez-Lima, 2011, Sivapathasuntharam et al., 2017, Weinrich et al., 2017). This, in turns, triggers a cascade of secondary downstream signaling pathways that collectively stimulate intrinsic neuroprotective mechanisms (Hamblin, 2016, Khan and Arany, 2015, Rojas and Gonzalez-Lima, 2011). With regard to our findings here, our long-term PBM may not have been efficient in activating these beneficial mechanisms and to prevent neuronal loss in aging, at least among the Pv^+^ cells of the striatum. Perhaps a different dosage may have been more efficient; for various neurological disorders, from Alzheimer's to Parkinson's disease, dosage has been reported to be important in effectiveness of PBM as a neuroprotective agent (Hamblin, 2016, Khan and Arany, 2015, Rojas and Gonzalez-Lima, 2011), and the same may be the case in aging.

Our analysis of the dopaminergic system, in terms of terminals in the striatum and cell bodies in the midbrain SNc, indicated that it remained stable in mice aged 12 months. These findings are consistent with previous ones in animals of this age (Schumm et al., 2012). Furthermore, our results indicated that, as with the striatal interneurons, long-term PBM had no deleterious effects on these key structures of the basal ganglia. After 8 months of daily exposure, the density of dopaminergic terminations and the number of their cell bodies in the 12m and 12m + PBM groups were near identical (Fig. 3). This result has positive implications for the safety of long-term PBM on the dopaminergic neurons (and striatal interneurons) and for future use in Parkinson's disease patients (Mitrofanis, 2017).

In conclusion, our results indicated that long-term PBM had beneficial effects on the aging brain; this treatment was effective in reducing glial cell number and did not have any deleterious effects on the neurons and terminations in the striatum; and that this treatment was not toxic to these cells after such a long-term exposure in the aged brain is a key safety issue, particularly when considering use in humans. Future studies may explore any functionally related changes, for example, using molecular and/or electrophysiological methods, in the aging striatum and if there is any impact on these changes after PBM.

## Disclosure statement

There are no conflicts of interests to declare.

## References

[bib1] Bakalian A., Corman B., Delhaye-Bouchaud N., Mariani J. (1991). Quantitative analysis of the Purkinje cell population during extreme ageing in the cerebellum of the Wistar/Louvain rat. Neurobiol. Aging.

[bib2] Balaban R.S., Nemoto S., Finkel T. (2005). Mitochondria, oxidants, and aging. Cell.

[bib3] Beach T.G., Walker R., McGeer E.G. (1989). Patterns of gliosis in Alzheimer’s disease and aging cerebrum. Glia.

[bib4] Begum R., Powner M.B., Hudson N., Hogg C., Jeffery G. (2013). Treatment with 670 nm light up regulates cytochrome C oxidase expression and reduces inflammation in an age-related macular degeneration model. PLoS One.

[bib6] Brahmachari S., Fung Y.K., Pahan K. (2006). Induction of glial fibrillary acidic protein expression in astrocytes by nitric oxide. J. Neurosci..

[bib7] Chaturvedi R.K., Beal M.F. (2008). Mitochondrial approaches for neuroprotection. Ann. N. Y. Acad. Sci..

[bib8] Conde J.R., Streit W.J. (2006). Microglia in the aging brain. J. Neuropathol. Exp. Neurol..

[bib9] Cotrina M.L., Nedergaard M. (2002). Astrocytes in the aging brain. J. Neurosci. Res..

[bib10] Damani M.R., Zhao L., Fontainhas A.M., Amaral J., Fariss R.N., Wong W.T. (2011). Age-related alterations in the dynamic behavior of microglia. Aging Cell.

[bib11] Darbin O. (2012). The aging striatal dopamine function. Parkinsonism Relat. Disord..

[bib12] Eells J.T., Wong-Riley M.T.T., VerHoeve J., Henry M., Buchman E.V., Kane M.P., Gould L.J., Das R., Jett M., Hodgson B.D., Margolis D., Whelan H.T. (2004). Mitochondrial signal transduction in accelerated wound and retinal healing by near-infrared light therapy. Mitochondrion.

[bib13] El Massri N., Cullen K.M., Stefani S., Moro C., Torres N., Benabid A.-L., Mitrofanis J. (2018). Evidence for encephalopsin immunoreactivity in interneurones and striosomes of the monkey striatum. Neurosci. Res..

[bib14] El Massri N., Johnstone D.M., Peoples C.L., Moro C., Reinhart F., Torres N., Stone J., Benabid A.-L., Mitrofanis J. (2016). The effect of different doses of near infrared light on dopaminergic cell survival and gliosis in MPTP-treated mice. Int. J. Neurosci..

[bib15] El Massri N., Moro C., Torres N., Darlot F., Agay D., Chabrol C., Johnstone D.M., Stone J., Benabid A.-L., Mitrofanis J. (2016). Near-infrared light treatment reduces astrogliosis in MPTP-treated monkeys. Exp. Brain Res..

[bib16] Fabricius K., Jacobsen J.S., Pakkenberg B. (2013). Effect of age on neocortical brain cells in 90+ year old human females—a cell counting study. Neurobiol. Aging.

[bib17] Fernagut P.O., Diguet E., Bioulac B., Tison F. (2004). MPTP potentiates 3-nitropropionic acid-induced striatal damage in mice: reference to striatonigral degeneration. Exp. Neurol..

[bib18] Gkotsi D., Begum R., Salt T., Lascaratos G., Hogg C., Chau K.-Y., Schapira A.H.V., Jeffery G. (2014). Recharging mitochondrial batteries in old eyes. Near infra-red increases ATP. Exp. Eye Res..

[bib19] Hamblin M.R. (2016). Shining light on the head: photobiomodulation for brain disorders. BBA Clin..

[bib20] Karu T. (2010). Mitochondrial mechanisms of photobiomodulation in context of new data about multiple roles of ATP. Photomed. Laser Surg..

[bib21] Kawaguchi Y., Wilson C.J., Augood S.J., Emson P.C. (1995). Striatal interneurones: chemical, physiological and morphological characterization. Trends Neurosci..

[bib22] Khan I., Arany P. (2015). Biophysical approaches for oral wound healing: emphasis on photobiomodulation. Adv. Wound Care (New Rochelle).

[bib23] Kujoth G.C., Hiona A., Pugh T.D., Someya S., Panzer K., Wohlgemuth S.E., Hofer T., Seo A.Y., Sullivan R., Jobling W.A., Morrow J.D., Van Remmen H., Sedivy J.M., Yamasoba T., Tanokura M., Weindruch R., Leeuwenburgh C., Prolla T.A. (2005). Mitochondrial DNA mutations, oxidative stress, and apoptosis in mammalian aging. Science.

[bib24] Linnane A.W., Marzuki S., Ozawa T., Tanaka M. (1989). Mitochondrial DNA mutations as an important contributor to ageing and degenerative diseases. Lancet.

[bib25] Lynch A.M., Murphy K.J., Deighan B.F., O’Reilly J.-A., Gun’ko Y.K., Cowley T.R., Gonzalez-Reyes R.E., Lynch M.A., Lynch A.M., Murphy K.J., Deighan B.F., O’Reilly J.-A., Gun’ko Y.K., Cowley T.R., Gonzalez-Reyes R.E., Lynch M.A. (2014). The impact of glial activation in the aging brain. Aging Dis..

[bib26] Ma W., Wong W.T. (2016). Aging changes in retinal microglia and their relevance to age-related retinal disease. Adv. Exp. Med. Biol..

[bib27] Mouton P.R., Long J.M., Lei D.-L., Howard V., Jucker M., Calhoun M.E., Ingram D.K. (2002). Age and gender effects on microglia and astrocyte numbers in brains of mice. Brain Res..

[bib44] Mitrofanis J. (2017). Why and how does lighttherapy offer neuroprotection in Parkinson’s disease?. Neural. Regen. Res..

[bib28] Ohm T.G., Busch C., Bohl J. (1997). Unbiased estimation of neuronal numbers in the human nucleus coeruleus during aging. Neurobiol. Aging.

[bib29] Parent A., Hazrati L.-N. (1995). Functional anatomy of the basal ganglia. I. The cortico-basal ganglia-thalamo-cortical loop. Brain Res. Rev..

[bib45] Paxinos G., Franklin K. (2001). The mouse brain in stereotaxic coordinates.

[bib30] Pelvig D.P., Pakkenberg H., Stark A.K., Pakkenberg B. (2008). Neocortical glial cell numbers in human brains. Neurobiol. Aging.

[bib31] Ramírez J.M., Ramírez A.I., Salazar J.J., de Hoz R., Triviño A. (2001). Changes of astrocytes in retinal ageing and age-related macular degeneration. Exp. Eye Res..

[bib32] Rojas J., Gonzalez-Lima F. (2011). Low-level light therapy of the eye and brain. Eye Brain.

[bib33] Salvadores N., Sanhueza M., Manque P., Court F.A. (2017). Axonal degeneration during aging and its functional role in neurodegenerative disorders. Front. Neurosci..

[bib34] Schumm S., Sebban C., Cohen-Salmon C., Callebert J., Launay J.-M., Golmard J.-L., Boussicault L., Petropoulos I., Hild A., Rousselet E., Prigent A., Friguet B., Mariani J., Hirsch E.C. (2012). Aging of the dopaminergic system and motor behavior in mice intoxicated with the parkinsonian toxin 1-methyl-4-phenyl-1,2,3,6-tetrahydropyridine. J. Neurochem..

[bib35] Shaw V.E., Spana S., Ashkan K., Benabid A.-L., Stone J., Baker G.E., Mitrofanis J. (2010). Neuroprotection of midbrain dopaminergic cells in MPTP-treated mice after near-infrared light treatment. J. Comp. Neurol..

[bib36] Sivapathasuntharam C., Sivaprasad S., Hogg C., Jeffery G. (2017). Aging retinal function is improved by near infrared light (670 nm) that is associated with corrected mitochondrial decline. Neurobiol. Aging.

[bib37] Soreq L., Rose J., Soreq E., Hardy J., Trabzuni D., Cookson M.R., Smith C., Ryten M., Patani R., Ule J. (2017). Major shifts in glial regional identity are a transcriptional hallmark of human brain aging. Cell Rep..

[bib38] Tremblay M.-È., Zettel M.L., Ison J.R., Allen P.D., Majewska A.K. (2012). Effects of aging and sensory loss on glial cells in mouse visual and auditory cortices. Glia.

[bib39] Unger J.W. (1998). Glial reaction in aging and Alzheimer’s disease. Microsc. Res. Tech..

[bib40] Vacano G.N., Gibson D.S., Turjoman A.A., Gawryluk J.W., Geiger J.D., Duncan M., Patterson D. (2018). Proteomic analysis of six- and twelve-month hippocampus and cerebellum in a murine Down syndrome model. Neurobiol. Aging.

[bib41] Weinrich T.W., Coyne A., Salt T.E., Hogg C., Jeffery G. (2017). Improving mitochondrial function significantly reduces metabolic, visual, motor and cognitive decline in aged *Drosophila melanogaster*. Neurobiol. Aging.

[bib42] Zecha J.A.E.M., Raber-Durlacher J.E., Nair R.G., Epstein J.B., Sonis S.T., Elad S., Hamblin M.R., Barasch A., Migliorati C.A., Milstein D.M.J., Genot M.-T., Lansaat L., van der Brink R., Arnabat-Dominguez J., van der Molen L., Jacobi I., van Diessen J., de Lange J., Smeele L.E., Schubert M.M., Bensadoun R.-J. (2016). Low level laser therapy/photobiomodulation in the management of side effects of chemoradiation therapy in head and neck cancer: part 1: mechanisms of action, dosimetric, and safety considerations. Support Care Cancer.

